# STING-mediated neuroinflammation: a therapeutic target in neurodegenerative diseases

**DOI:** 10.3389/fnagi.2025.1659216

**Published:** 2025-09-19

**Authors:** Haoqin Zhang, Ziyang He, Can Yin, Shiyi Yang, Jianqiao Li, Hong Lin, Guishan Hu, Anguo Wu, Dalian Qin, Guangqiang Hu, Lu Yu

**Affiliations:** ^1^Sichuan Key Medical Laboratory of New Drug Discovery and Druggability Evaluation, School of Pharmacy, School of Basic Medical Sciences, Southwest Medical University, Luzhou, China; ^2^Department of Medical Imaging, Southwest Medical University, Luzhou, Sichuan, China; ^3^Clinic Medical College, Southwest Medical University, Luzhou, Sichuan, China; ^4^Integrated Traditional Chinese and Western Medicine College, Southwest Medical University, Luzhou, Sichuan, China; ^5^Department of Anatomy, School of Basic Medical Sciences, Southwest Medical University, Luzhou, China

**Keywords:** stimulator of interferon genes, neuroinflammation, neurodegenerative diseases, cGAS-STING signal pathway, modulators, therapeutic strategies

## Abstract

The stimulator of interferon genes (STING) plays a crucial role as an adaptor in innate immune defense, orchestrating key inflammatory processes through the modulation of type I interferon signaling and activation of cytokine networks. Recent studies have identified STING-induced neuroinflammatory responses as a major factor in the progression of neurological diseases, particularly in neurodegenerative disorders. This review methodically explores the structural basis of STING activation and its role in driving pathological inflammation. And the classic and non-classic pathways of STING as well as their roles in neurodegenerative diseases were discussed. Additionally, it critically assesses new pharmacological approaches that target the STING pathway, emphasizing anti-inflammatory treatments ranging from synthetic small-molecule inhibitors to bioactive compounds sourced from traditional Chinese medicines, which aim to mitigate neurotoxic inflammation. By combining mechanistic insights with therapeutic advancements, this paper presents an innovative transformation framework aimed at developing anti-inflammatory therapies targeting the STING pathway to treat neurodegenerative diseases. The core contribution of this framework lies in systematically bridging the innate immune regulation and neuroinflammation control mechanisms, providing a new strategy for disease intervention.

## Graphical Abstract

**Figure:**
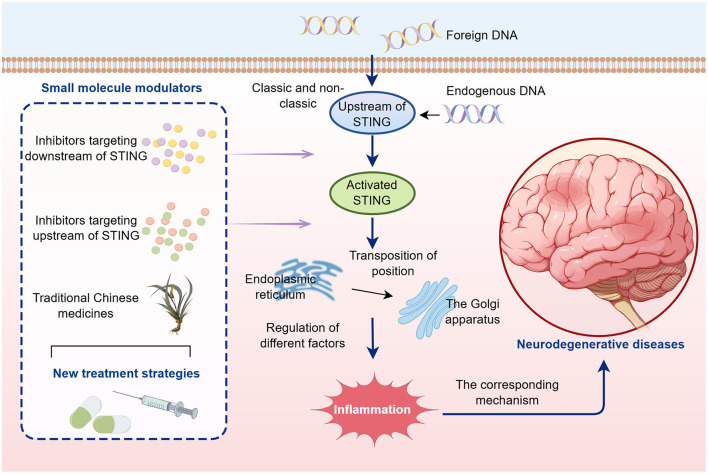

## 1 Introduction

Conventionally, DNA has long been regarded as immunologically inert. However, some researchers that made a groundbreaking discovery in the field of antiviral immunity research by unveiling the pivotal role played by the stimulator of STING, in inducing innate immune responses triggered by intracellular DNA (Ishikawa and Barber, 2008; Zhong et al., 2008; Sun et al., 2009). Upon binding with cyclic GMP-AMP (cGAMP) and other cyclic dinucleotides (CDNs), cytosolic double-stranded DNA (dsDNA) of diverse origins transmit the intracellular signal to STING's resident proteins in the endoplasmic reticulum (ER). This biochemical interaction induces conformational changes in STING's C-terminus, resulting in ER-to-Golgi transport via vesicular mechanisms (Shang et al., 2019). This sophisticated biological cascade involves multiple signaling networks, comprising both the classical Cyclic GMP-AMP synthase (cGAS)-STING axis and alternative STING-activation routes mediated by additional molecular detectors (Paul et al., 2021). Neurodegenerative disorders constitute a clinically and pathologically diverse spectrum of conditions characterized by the gradual deterioration of neuronal populations and selective vulnerability of specific neural circuits (Agnello and Ciaccio, 2022). The STING signaling axis and its downstream cascades are involved in disease pathogenesis. In recent years, pharmacological modulation of this pathway has emerged as a promising therapeutic strategy for neurodegenerative disorders. This review systematically elucidated the critical roles of STING and its associated pathways in various neurodegenerative diseases (NDs) while also exploring regulators of the different mechanisms of the STING pathway, and incorporate traditional Chinese herbal medicines (Graphical abstract). We anticipated that this comprehensive review would offer novel insights into targeting STING for effective treatment of NDs and serve as a favorable guide for developing innovative therapeutic strategies.

## 2 Overview of STING proteins

### 2.1 The basic structure of STING

**Figure 1:**
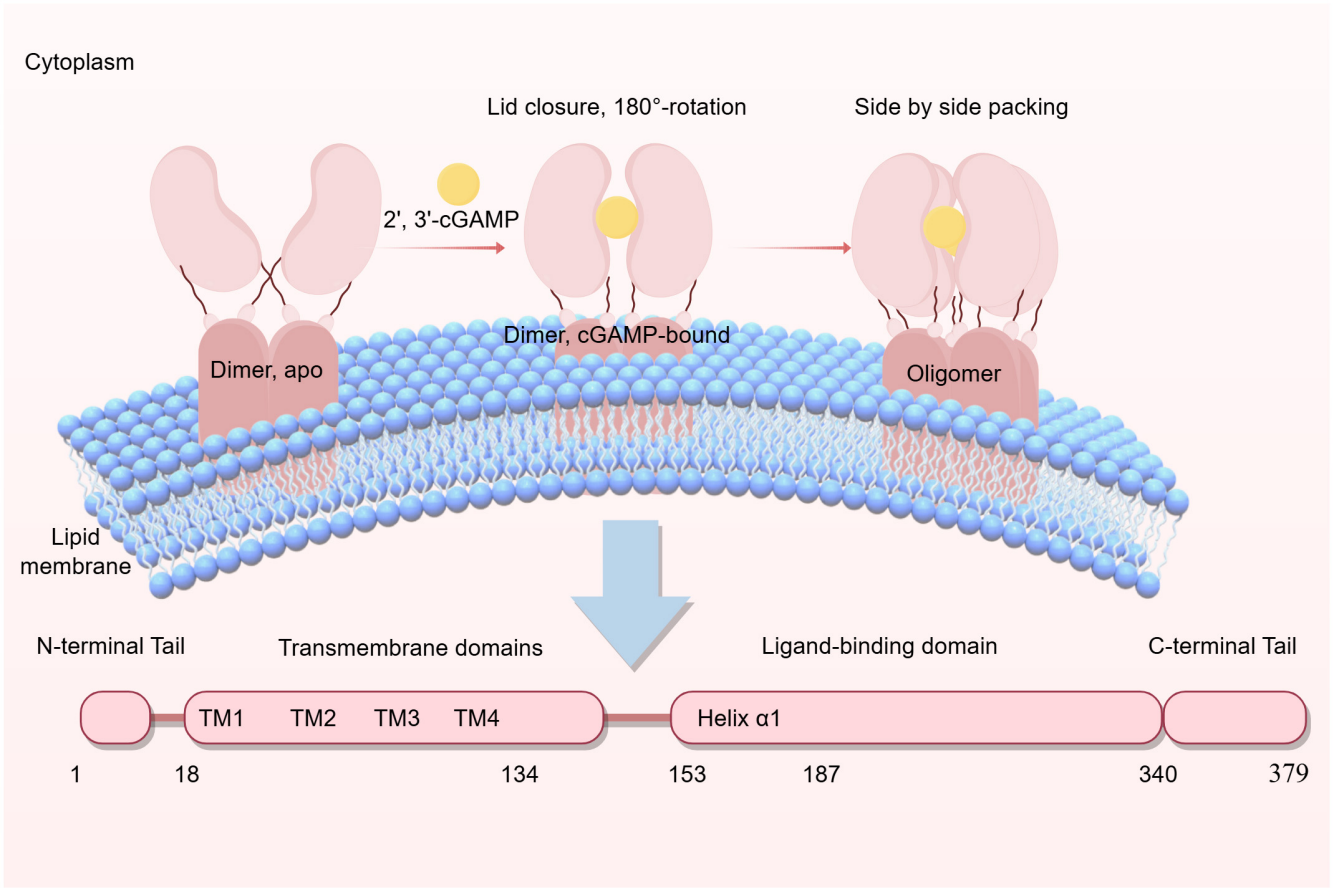
The structure of STING begins with the N-terminal tail, and its four transmembrane domains (TM1–TM4) anchor it to the endoplasmic ER membrane or other organelle membranes, enabling inter-organelle trafficking. The connector helix (Helix α1) between TM4 and the LBD is crucial for the folding, dimerization, and binding of cyclic di-GMP. The LBD specifically binds bacterial-derived cyclic di-GMP and host-derived2′3′-cGAMP, forming a butterfly-shaped dimer that activates STING. CTT plays a pivotal role after STING activation, recruiting TBK1 and IRF3 to initiate downstream immune responses. Upon binding of cGAMP to the LBD, STING undergoes conformational changes, exposing the CTT and facilitating its transport from the ER to the Golgi apparatus via COP-II vesicles. This process is a critical step in STING signaling activation. Together, these structural changes and trafficking processes coordinate the core functions of STING in innate immune responses.

The human STING polypeptide consists of 379 amino acid residues, exhibiting a molecular mass of roughly 42 kilodaltons (Shang et al., 2019). The architectural configuration of STING comprises four distinct structural domains: a condensed amino-terminal cytoplasmic segment (residues 1–17); four transmembrane-spanning α-helical motifs (residues 18–134) providing membrane integration with the ER; a cytoplasmic ligand recognition module (residues 153–340) responsible for signal transduction initiation; and a carboxy-terminal regulatory element (residues 340–379) that facilitates distal signaling cascades through coordinated engagement with TANK-binding kinase 1 (TBK1; Zhang et al., 2020). The initial structural module encompasses four N-terminal transmembrane α-helical motifs (TM1–4), functionally dedicated to lipid bilayer integration and orchestrating vesicular transport dynamics (Shang et al., 2019). The α1-helix linking TM4 to the ligand-binding domain (LBD)'s core helix (residues 153–177) is essential for proper folding and dimer formation. Disruption of this helical structure triggers cytoplasmic STING aggregation and dimer dissociation through impaired hydrophobic dimer interfaces (Huang et al., 2012). Ouyang et al. (2012) demonstrated that the α1-helix mediates stable intermolecular hydrogen-bond networks with cyclic di-GMP across STING dimers, establishing its essential mechanistic involvement in diverse signaling activities. The carboxy-terminal region (residues 174–379) constitutes the cytoplasmically localized soluble domain, comprising an LBD coupled with a C-terminal tail (CTT). The LBD operates as a structural dimer, functioning as the minimal catalytic module for 2′3′-cGAMP and CDN recognition. Its architecture integrates an α/β-fold topology with five β-sheets and four α-helices. Helices α1–3 drive dimeric assembly, generating a bilobed architecture. All CDNs universally assume a U-shaped geometry, engaging the conserved interfacial cleft at the dimeric STING junction through stereospecific interactions. CTT, spanning ~40 residues, occupies the terminal end of the CDN-binding domain in both human and murine STING orthologs, harboring the IRF3 docking interface (de Oliveira Mann et al., 2019). CTT mainly exists in the form of dimers in solution (Ergun et al., 2019), which protects the STING polymer interface from self-activation and recruits interferon regulators and TBK1 (Burdette et al., 2011). The structural asymmetry of cGAMP enables dual binding orientations corresponding to the two distinct oblique interfaces observed in guanine- or adenine-bound STING dimers. This molecular asymmetry permits simultaneous engagement with two symmetric STING dimers in antiparallel configurations (Shi et al., 2015). The STING dimer features complementary electrostatic surfaces with interdomain stabilization through polar contacts. cGAMP anchoring involves dual hydrogen bonding with Tyr167 and Arg238, establishing planar stacking with purine bases. These interactions engage Asn7 and the guanidine moiety, respectively, while extensive hydrogen-bonding networks—including water-bridged contacts—reinforce ligand positioning along the STING interface (Pan et al., 2023). The cGAMP phosphate backbone and ribose hydroxyl groups are stabilized by an extensive hydrogen-bond network. Strategically positioned residues beneath the ligand plane facilitate high-affinity cGAMP engagement with the STING dimer. The STING-cGAMP complex adopts a bilobed quaternary structure, with the LBD oriented antiparallel to the TM domain, thereby exposing CTT (Shang et al., 2019; Ergun et al., 2019). This structural rearrangement potentially promotes the binding of COP-II coat proteins, thereby mediating STING's ER export and subsequent vesicular trafficking through the secretory pathway to reach both the ER-Golgi intermediate compartment and Golgi complex (ERGIC; Dobbs et al., 2015; Gui et al., 2019; Prabakaran et al., 2018). The basic structure and related conformations of the STING protein are shown in Figure 1.

## 3 The molecular mechanism involving STING

### 3.1 The classical STING pathway: cGAS-STING

**Figure 2:**
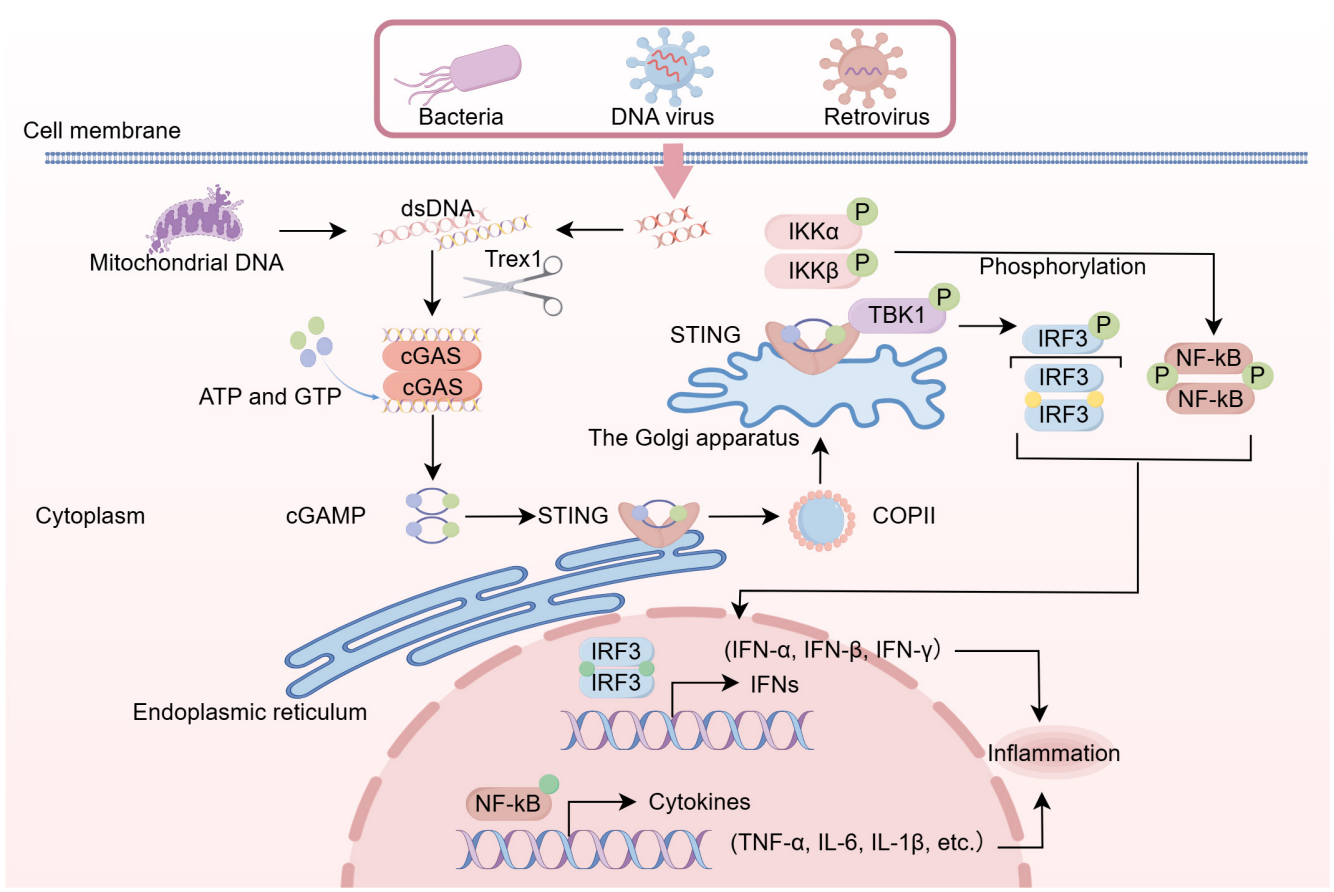
DNA from diverse origins, including mitochondrial, viral, and bacterial DNA, can initiate the activation of cGAS. cGAS catalyzes the synthesis of cGAMP, which serves as a secondary messenger to activate STING. The activation of STING leads to the recruitment and activation of TBK1, subsequently phosphorylating IRF3, which dimerizes and translocates to the nucleus, inducing the expression of IFNs and thereby triggering a DNA-driven immune response. Moreover, the STING pathway also facilitates the production of inflammatory cytokines via the NF-κB pathway. The activated STING pathway plays a crucial role in the host's inflammatory response.

cGAS functions as a critical upstream activator of the STING signaling pathway (Decout et al., 2021). The 60-kilodalton cGAS detects cytosolic dsDNA of diverse origins, encompassing viral, bacterial, mitochondrial, and retrotransposon-derived sequences (Gan et al., 2021). The cGAS protein consists of three distinct structural domains: an N-terminal DNA-binding domain (Kranzusch et al., 2013), a central nucleotidyltransferase catalytic domain, and a C-terminal functional domain. Upon recognition of dsDNA, cGAS undergoes intermolecular cross-linking, forming dimers or oligomers. Upon cGAS-dsDNA complex formation, ATP and GTP undergo cyclization via dual phosphodiester bond formation, yielding 2′3′-cGAMP as the STING-activating secondary messenger (Decout et al., 2021). Following STING activation, adaptor dimerization enables Tripartite Motif Containing 56-catalyzed ubiquitination, promoting TBK1 recruitment (Shu et al., 2012; Tsuchida et al., 2010). This trafficking cascade mediates STING translocation from the ER through the ERGIC and Golgi apparatus to post-Golgi vesicles (Chen et al., 2016; Li and Chen, 2018). Coat Protein Complex II vesicle formation requires the coordinated assembly of coatomer proteins, notably Sar1, Sec13, Sec24, and Sec31 (Gui et al., 2019; Sun et al., 2022). Activated STING moves from the ER to the Golgi apparatus, where it recruits TBK1 and IκB kinase ε. These kinases phosphorylate STING, then activate IRF3 and NF-κB, ultimately triggering the expression of antimicrobial cytokines (Tanaka and Chen, 2012; Abe and Barber, 2014; Luo et al., 2016; Zhang et al., 2019; Balka et al., 2020). Concurrently, the phosphorylated STING domain facilitates TBK1-IRF3 complex formation, mediating TBK1-dependent IRF3 phosphorylation (Zhang et al., 2019). Following dimer formation, the activated IRF3 complex migrates into the nuclear compartment, where it initiates transcriptional activation of type I interferons (IFN-α/β). This cascade subsequently upregulates interferon-inducible gene networks, ultimately coordinating host antiviral defense mechanisms (Hopfner and Hornung, 2020). This process stimulates NF-κB activation, subsequently enhancing IFN-β synthesis and other inflammatory or antiviral cytokine secretion (Jeffries and Marriott, 2020). STING-deficient cells exhibit impaired recognition of DNA viruses and diminished pro-inflammatory responses to synthetic DNA ligands. Furthermore, STING induces autophagy through canonical (ULK/TBK1-dependent) and non-canonical (WIPI2/ATG5-12-16L1-mediated) mechanisms (Govindarajulu et al., 2023). Following oligomerization, STING-enriched ERGIC membranes function as critical platforms for LC3 lipidation, thereby promoting the biogenesis of autophagosomes. The subsequent fusion with lysosomes enables the degradation of cellular cargo (Gui et al., 2019). Figure 2 shows the basic signaling pathway of cGAS-STING.

### 3.2 The non-classical signaling pathway of STING

**Figure 3:**
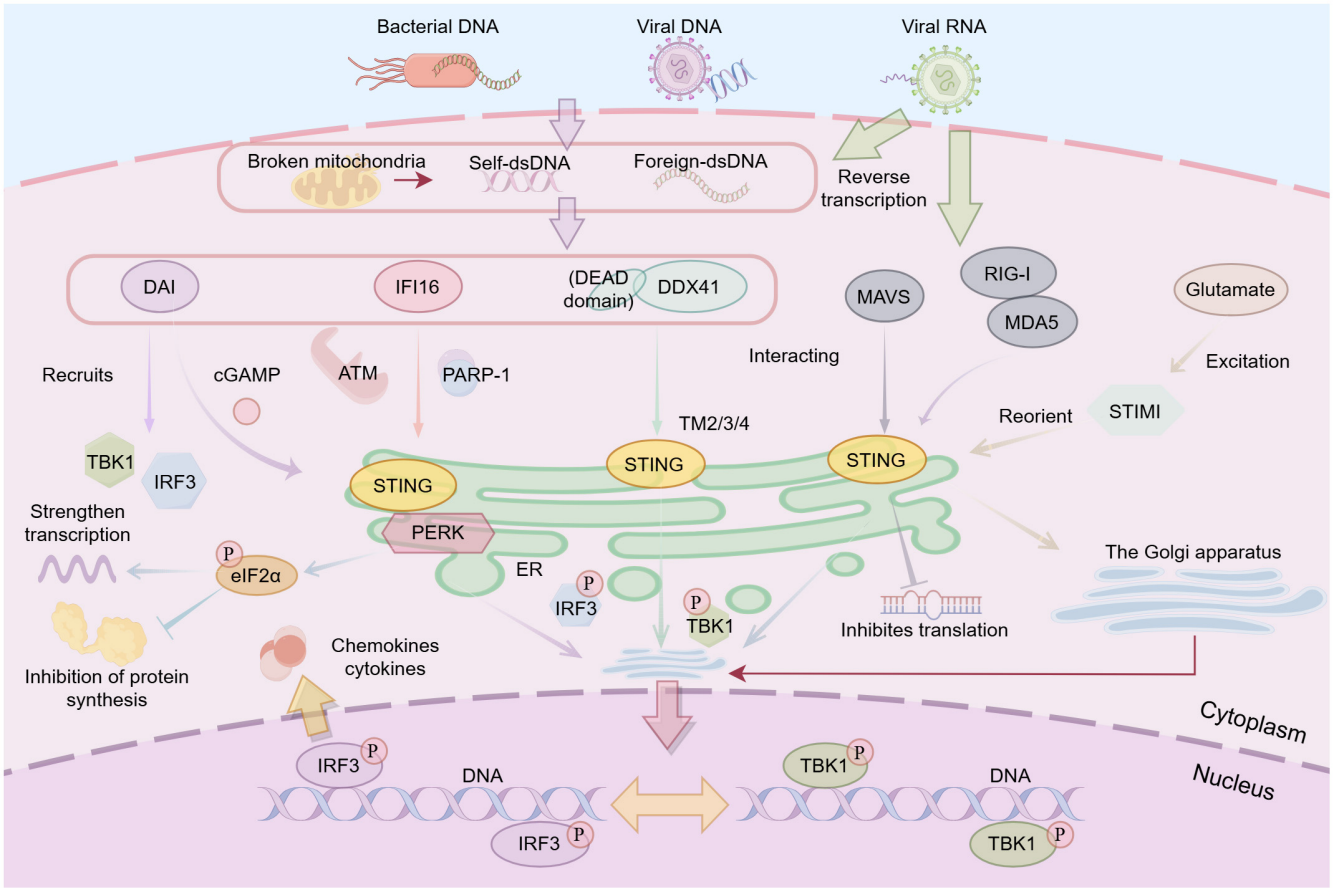
Host dsDNA and mtDNA are recognized by specific sensor proteins: DAI, IFI16, and DDX41 are responsible for detecting DNA, while RIG-I and MDA5 identify RNA, transmitting signals through MAVS. Upon recognition, DAI catalyzes the synthesis of cGAMP, which subsequently activates STING protein. The DNA damage response is regulated by ATM and PARP-1 during this process. Additionally, DDX41 interacts with STING through its transmembrane domains TM2/3/4, while RIG-I and MDA5 facilitate the translocation of STING from the ER to the Golgi apparatus. Excitatory glutamate also facilitates STING transport from the ER to the Golgi apparatus by reorienting STIMI. Autophagy-mediated degradation of STING is also crucial in preventing excessive inflammatory responses. Ultimately, STING recruits TBK1, which phosphorylates IRF3, induces IRF3 dimerization and translocation into the nucleus, where it initiates the transcription of type I interferons, chemokines, and cytokines, thereby orchestrating the immune response. PERK is located on the endoplasmic reticulum and, after interacting with STING, promotes the phosphorylation of eIF2α through PERK-mediated phosphorylation, thereby inhibiting protein production and enhancing the transcription of specific mRNAs.

As a pattern recognition receptor (PRR), STING can be activated by CDNs or cGAMP (Burdette et al., 2011; Dey et al., 2015). Beyond the canonical cGAS-STING axis, STING exhibits alternative activation mechanisms, as outlined below. Cytosolic DNA sensors identified to date include the DNA-dependent activator of interferon-regulatory factors (DAI; Takaoka et al., 2007), IFNγ-inducible protein 16 (IFI16; Unterholzner et al., 2010), DEAD-Box Helicase 41 (DDX41; Zhang et al., 2011), and components of the RNA-sensing pathways. Figure 3 is a summary of each non-classical pathway of STING.

The DAI serves as a cytosolic DNA sensor initiating innate immune responses. DAI recruits both TBK1 serine/threonine kinase and IFN regulatory factor 3 (IRF3), essential for IFN-I and related gene activation (Takaoka et al., 2007). DAI detection of cytosolic DNA triggers cGAMP synthesis, which subsequently activates ER-localized STING (Kong et al., 2021). IFI16 is a member of the PYRIN and HIN200 domain-containing (PYHIN) protein family (Gasser et al., 2017). Etoposide rapidly induces NF-κB-mediated innate immunity via IFI16-dependent and STING-dependent, but cGAS/cGAMP-independent, DNA sensing. Pharmaceutical agents that induce genomic instability can trigger alternative STING pathway stimulation, leading to the assembly of a unique STING macromolecular signaling platform (Dunphy et al., 2018). In IFI16-knockout THP1 cells, etoposide fails to induce IFN-β transcription, while cGAS-deficient cells maintain normal induction (Jønsson et al., 2017). The DNA damage response proteins Ataxia Telangiectasia Mutated (ATM) and PARP-1 collaborate with IFI16 to facilitate non-canonical STING activation, leading to assembly of a distinct STING complex that incorporates p53 and TRAF6 (Dunphy et al., 2018). The DEXDc helicase DDX41 functions as a myeloid dendritic cell DNA sensor, with direct DNA binding to its DEAD domain and interaction with STING transmembrane domains (TM2-4; Zhang et al., 2011). In THP-1 cells, DDX41 constitutively associates with STING and initiates IFN-β production prior to IFI16 involvement, indicating its predominant role in early microbial DNA detection and rapid IFN-I induction.

The mitochondrial antiviral signaling protein (MAVS), alternatively termed VISA, Cardif or IPS-1, serves as an RNA-responsive adaptor that engages with and stimulates STING (Li et al., 2017). Enveloped RNA viruses (e.g., IAV) activate a cGAS-independent STING pathway via membrane fusion. Crucially, these viruses employ evasion strategies—such as the IAV HA2 fusion peptide binding STING directly—to inhibit dimerization and TBK1 activation. This highlights STING's role beyond canonical DNA sensing (Holm et al., 2016). Following RIG-I/MDA5 recognition of RNA ligands, RIG-I-like receptors-mediated STING activation induces viral translation suppression, independent of observable STING translocation or phosphorylation—analogous to DNA damage response pathways (Franz et al., 2018).

A recent investigation of ferroptosis, an iron-mediated cell death pathway, revealed neuronal STING expression during neuroinflammation. ER retention via Stromal Interaction Molecule 1 (STIM1) binding maintains STING in an inactive state until glutamate excitation triggers STIM1 translocation, enabling STING Golgi trafficking and activation. Notably, neurons exhibit transport-dependent STING activation that bypasses canonical dsDNA ligand recognition (Woo et al., 2024). Crucially, persistent STIM1-STING signaling acts via a TBK1/IRF3-independent pathway to trigger autophagic degradation of glutathione peroxidase 4 (GPX4)—the central regulator preventing lipid peroxidation in ferroptosis. This axis drives iron-dependent ROS accumulation, ultimately inducing neuronal ferroptosis (Ingold et al., 2018).

In addition to triggering the secretion of TBK1 and IFN through the IRF3 or NF-κB pathways, as well as the release of various pro-inflammatory factors, STING still has non-classical downstream activation pathways. Protein kinase RNA-like ER kinase (PERK) is an ER-transmembrane kinase that senses abnormal conditions within the ER. It stops the protein translation process to prevent protein misfolding (Hetz, 2012). Zhang et al. (2022a) visualized the interaction between PERK and STING after immunoprecipitation using proximity ligation assay and domain mapping assay. Mechanistically, the fusion of the C-terminal domain of STING continuously enhances the auto-activation ability of the kinase domain of PERK. After cGAMP activation in the PERK-eukaryotic initiation factor 2α (eIF2α) pathway, STING can bind to PERK, promoting eIF2α phosphorylation mediated by PERK. Its activation usually hinders the production of proteins and particularly enhances the transcription of specific mRNAs, thereby leading to the development of various diseases.

The molecular basis of context-dependent STING pathway stimulation and its functional implications in STING-mediated signaling requires further elucidation. Current evidence regarding neuronal STING pathways beyond canonical cGAS-STING signaling remains incomplete, warranting additional studies to assess their potential relevance to neurodegenerative disorders.

## 4 Regulation of STING in neurodegenerative diseases

**Figure 4:**
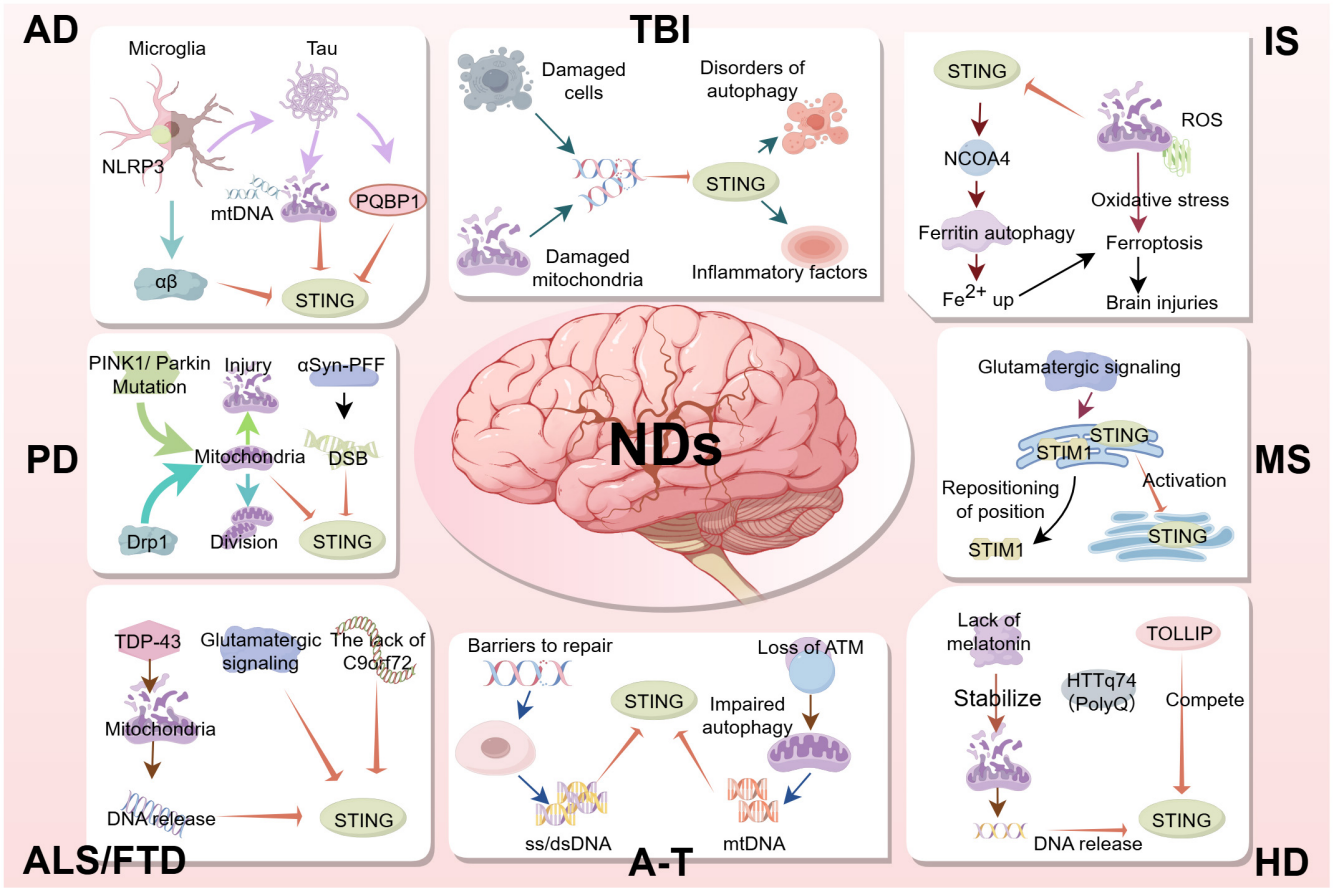
In AD, the activation of STING in microglia exacerbates cognitive decline through neuroinflammation and Aβ/tau pathology. In PD, mitochondrial dysfunction and the release of mtDNA activate STING, resulting in the loss of dopaminergic neurons and motor deficits. In ALS and FTD, the accumulation of TDP-43 and the release of mtDNA trigger STING-mediated inflammation, driving motor neuron degeneration. In A-T, defects in DNA repair and mtDNA release activate STING, leading to neuroinflammation and premature aging. In HD, the release of mtDNA and oxidative stress activate STING, further worsening neurodegeneration and motor dysfunction. In MS, the activation of STING in neurons and microglia promotes neuroinflammation and demyelination. In IS, mitochondrial damage and mtDNA release activate STING, exacerbating ischemic brain injury and inflammation. In TBI, STING activation mediates neuroinflammation and autophagy dysfunction, resulting in secondary brain injury. These findings suggest that STING may represent a potential therapeutic target for NDs.

NDs encompass progressive central nervous system (CNS) disorders characterized by neuronal degeneration and myelin pathology, resulting in gradual cognitive/behavioral impairment. NDs are classified as chronic or acute. The former mainly includes Alzheimer's disease (AD), Parkinson's disease (PD), Amyotrophic lateral sclerosis (ALS), Frontotemporal dementia (FTD), Ataxia telangiectasia (A-T), Huntington's disease (HD), Multiple sclerosis (MS), and the latter mainly includes ischemia stroke (IS) and traumatic brain injury (TBI). Although classified as an immune disorder, Aicardi-Goutieres Syndrome (AGS) is also classified as a neurological lesion because it mainly presents in the nervous system. Neuroinflammation involves microglia, resident brain cells, and occasionally peripherally-derived immune cells, which collectively release cytokines and chemokines with dual neuroprotective and neurotoxic effects (Kwon and Koh, 2020). Mitochondrial reactive oxygen species (mtROS) represent pivotal mediators of NOD-like Receptor Family Pyrin Domain Containing 3 (NLRP3) inflammasome activation, driving neuroinflammatory pathogenesis (Dong et al., 2021; Quan et al., 2020). And factors such as oxidative stress and DNA damage release are associated with the STING pathway. STING signaling triggers cellular senescence following DNA damage, thereby activating upstream inflammatory pathways and exacerbating tissue injury (Chen et al., 2018; Dou et al., 2017). STING additionally promotes proinflammatory amplification loops by facilitating intercellular communication through gap junction-mediated signaling between adjacent cells (Ablasser et al., 2013). Collectively, these findings demonstrate that the STING pathway plays a crucial role in NDs progression, though its activation mechanism varies across disease states. Therefore, regulating the STING signaling pathway represents a promising therapeutic strategy for NDs. Subsequently, we will delve into the specific role that STING plays in various prevalent NDs. The interaction mechanism between STING and various NDs is briefly summarized in Figure 4.

### 4.1 Role of STING signaling in Alzheimer's disease

AD manifests as progressive cognitive impairment, driven by amyloid-β (Aβ) plaque deposition and neurofibrillary tau pathology in susceptible brain regions (Hinkle et al., 2022). As resident phagocytes of the CNS, microglia cells primarily release degrading enzymes to degrade Aβ through degradation processes (Heneka, 2017). Sustained NLRP3 inflammasome signaling in microglia impairs their phagocytic function, compromising Aβ and neurofibrillary tangle clearance. This establishes a vicious cycle of pathological protein accumulation and chronic microglial activation (Tejera et al., 2019). Xie and colleagues demonstrated cGAS activation through cytosolic dsDNA binding in AD patient brains. In 5xFAD models, phospho-STING/IRF3 co-localization with CD68+ microglia adjacent to Aβ plaques in the dentate gyrus confirmed microglial cGAS-STING pathway engagement (Xie et al., 2023). cGAS knockdown ameliorated cognitive deficits, attenuated Aβ pathology, and suppressed interferon-stimulated gene (ISG) expression in the hippocampus. These findings indicate that the cGAS-STING axis regulates neuroinflammation via crosstalk among microglia, astrocytes, and neurons. Intracellular aggregates of misfolded tau protein represent another pathological hallmark of AD. In general, microglia take up tau through two surface receptors, LRP1 and TREM2. The DNA sensor Polyglutamine-binding protein 1 directly engages tau3R/4R isoforms, subsequently initiating cGAS-STING-mediated innate immune activation (Jin et al., 2021). The human Tau isoforms 410 and 441 exhibit intrinsic potential to stimulate the cGAS-STING signaling cascade in mouse microglial cells, suggesting this mechanism may underlie multiple tauopathies such as Alzheimer's disease. A recent study explored this direction using AppNL-G-F/hTau-double knock-in (DKI) mice. The microglia in the brains of DKI mice exhibit typical neurodegenerative transcriptomic features, such as the downregulation of the homeostatic gene Tmem119 and the upregulation of clearance receptors (Trem2, Clec7a and Axl) and lipid metabolism (ApoE and Lpl), while the transcriptional levels of members of the cGAS-STING pathway continue to increase. Furthermore, pharmacological inhibition of STING was found to reduce brain inflammation and microglial synaptic phagocytosis. STING expression in both neurons and microglia was increased in the AD model mice. Inhibiting the activation of STING may serve as an effective therapeutic strategy for AD (Chung et al., 2024).

### 4.2 Role of STING signaling in Parkinson's disease

PD is neuropathologically characterized by Lewy pathology (Lewy bodies and neurites) accompanied by selective neuronal loss in the substantia nigra and vulnerable brain regions (Tolosa et al., 2021). PD patients and animal models exhibit sustained neuroinflammation, featuring microglial activation and increased pro-inflammatory cytokines that culminate in dopaminergic neuronal degeneration (Sampson et al., 2016; Gelders et al., 2018). Impaired PTEN-induced Kinase 1 (PINK1)/Parkin-dependent mitophagy leads to pathological mitochondrial accumulation, potentially driving PD pathogenesis (Newman and Shadel, 2018). PINK1 is a kinase that localizes to the mitochondria, while Parkin is a cytoplasmic E3 ubiquitin ligase (Shimura et al., 1999, 2000). Following PINK1 stabilization on the outer mitochondrial membrane (OMM), it phosphorylates both ubiquitin and Parkin's ubiquitin-like domain, triggering Parkin recruitment and subsequent OMM protein ubiquitination (Koyano et al., 2014). Following mitochondrial damage or uncoupling, Parkin translocates to the mitochondria and mediates the autophagic clearance of damaged mitochondria (Narendra et al., 2008). In Parkin-deficient mice, impaired mitophagy leads to mitochondrial DNA (mtDNA) leakage, activating cGAS-STING signaling and elevating pro-inflammatory cytokines and IFN-I (Sliter et al., 2018). The PINK1-Parkin mitophagy pathway maintains mitochondrial quality control by clearing damaged organelles, thus preventing cytoplasmic mtDNA accumulation and subsequent cGAS-STING hyperactivation that drives dopaminergic neurodegeneration. These findings definitively implicate the cGAS-STING-IFN-I signaling axis as a pivotal driver of neuroinflammatory processes and neurodegeneration in PD pathophysiology (Sliter et al., 2018). The STING protein interacts with misfolded α-synuclein, and subsequent cGAS-STING pathway activation triggers microglia-mediated dopaminergic neurotoxicity (Hinkle et al., 2022). In a primary neuronal culture model of PD exposed to αSyn-Pre-formed Fibrils (PFF), the induction of dsDNA breaks could be observed. The αSyn-PFF mouse model demonstrated early DNA damage and TBK1 activation in striatal microglia preceding dopaminergic neurodegeneration. Genetic ablation of STING in αSyn-PFF-treated mice attenuated IFN-I responses, improved motor function, reduced α-synuclein pathology, and preserved dopaminergic neurons (Hinkle et al., 2022). These findings reinforce the contribution of microglial activation and neuroinflammation to PD progression. Notably, pathogenic variants in leucine-rich repeat kinase 2 (LRRK2) represent a genetic link between familial and sporadic PD. In LRRK2-deficient macrophages, Drp1-mediated mitochondrial fragmentation and oxidative stress promote mtDNA release, leading to chronic cGAS-STING pathway activation (Li et al., 2022). Both Drp1 inhibition and antioxidant treatment mitigate this pathological cascade in LRRK2 knockout models. Furthermore, the participation of the cGAS-STING-YY1-Lipocalin 2 signaling cascade in controlling astrocyte senescence and the progression of PD has been determined. It has been identified that Lipocalin 2 is the effector of the cGAS-STING signaling, which is another new target for the treatment of PD (Jiang et al., 2023).

### 4.3 Role of STING signaling in amyotrophic lateral sclerosis/frontotemporal dementia

ALS is a progressive neurodegenerative disease characterized by the selective loss of upper and lower motor neurons in the brain and spinal cord, primarily affecting adults (Ragagnin et al., 2019), and it shares pathological features with about 50% of FTD cases (Bang et al., 2015). It affects the motor system, causing the degeneration of motor neurons in the brain's motor cortex except for the brain stem and spinal cord (Rowland and Shneider, 2001). About 4% of familial ALS patients have TAR DNA-binding Protein 43 (TDP-43) mutations (Sreedharan et al., 2008). The accumulation of TDP-43 in the cytoplasm is an important marker of ALS, which is associated with the characterization of neuroinflammatory cytokines in ALS patients, mainly manifested as NF-κB elevated levels and IFN-I pathways (Wang et al., 2011; Zhao et al., 2015). Several studies have now confirmed the presence of TDP-43 in mitochondria using model systems as well as neurons isolated postmortem from ALS patients (Davis et al., 2018; Ruan et al., 2017; Salvatori et al., 2018; Wang et al., 2019). Mitochondrial accumulation of TDP-43 triggers mtDNA leakage, stimulating the cGAS-STING axis and subsequent proinflammatory responses. This mechanism correlates with increased cGAMP concentrations observed in ALS patient spinal cord tissues (Yu et al., 2020). Research indicates TDP-43 enters mitochondria through the Translocase of Inner Mitochondrial Membrane 22 (TIM22) protein import machinery (Kumar and Julien, 2021). Experimental evidence confirms that neither wild-type nor Q331K mutant TDP-43 induces mtDNA release in TIM22-AGK-deficient cells (Yu et al., 2020). Hexanucleotide repeat expansions in C9orf72 represent the most prevalent genetic etiology of ALS. McCauley and colleagues demonstrated that C9orf72 deficiency triggers STING-dependent IFN-I production, driving neuroinflammation. Notably, STING inhibition attenuated pathological IFN-I responses in C9orf72^−/−^ murine models. Elevated IFN-I (IFN-I) signatures were detected in both circulating macrophages and CNS tissues from C9orf72-associated ALS/FTD patients. Pharmacological STING inhibition attenuated the augmented IFN-I response in patient-derived peripheral blood mononuclear cells (PBMCs; McCauley et al., 2020). Pathogenic variants in Superoxide Dismutase 1 (SOD1) underlie a subset of familial ALS cases. The SOD1-G93A transgenic mouse model recapitulates elevated IFN-I responses observed in human ALS. Importantly, cGAS/DDX41-STING-dependent IFN-I signaling exacerbates SOD1-mediated neurodegeneration, contributing to disease progression (Tan et al., 2022). Cortical hyperexcitability, a shared feature of ALS and FTD, may contribute to DNA damage through excessive glutamatergic neurotransmission (Benussi et al., 2017). Brief glutamate exposure (1 h) induced nuclear γH2AX accumulation and upregulated cytoplasmic STING expression. STING elevation was observed at both transcriptional and translational levels. These findings position STING inhibition as a promising therapeutic strategy for ALS.

### 4.4 Role of STING signaling in ataxia telangiectasia

A-T is an inherited neurodegenerative condition marked by aberrant cGAS-STING signaling, resulting in persistent neuroinflammation and autoimmune manifestations. Clinically, it may present as cerebellar ataxia, oculocutaneous telangiectasia, immunodeficiency, progressive respiratory insufficiency, and a higher susceptibility to malignancies (van Os et al., 2017). The ATM kinase serves as a master regulator of the DNA damage response pathway and is essential for preserving genomic stability. Defective DNA repair mechanisms can aberrantly activate the cGAS-STING inflammatory cascade (Paull, 2015). Song and colleagues proposed that in A-T, defective DNA repair results in cytoplasmic accumulation of single and dsDNA, which activates innate immunity through STING-mediated signaling. This pathway drives microglial activation and release of neurotoxic cytokines, representing a novel neuroinflammatory mechanism potentially relevant to multiple neurodegenerative disorders (Song et al., 2019). ATM deficiency impairs mitophagy, resulting in aberrant mtDNA leakage into the cytosol. Cytosolic mtDNA accumulation induces STING-dependent cellular senescence (Yang et al., 2021). Simultaneous knockout of ATM and STING reduced autoinflammatory phenotypes. The effect was more pronounced when cGAS was also eliminated in these mice (Härtlova et al., 2015). Aguado and colleagues demonstrated that H151, a selective STING antagonist, effectively suppressed self-DNA-triggered senescence-associated secretory phenotype activation and ameliorated neuropathological abnormalities in A-T brain organoids. Notably, cGAS-STING pathway inhibition conferred substantial neuroprotection and restored neuronal functionality in A-T models, implicating senescent astrocytes in mediating neuronal dysfunction. These findings align with established evidence of astrocyte-mediated neurotoxicity in neurodegenerative processes (Chinta et al., 2018). These results demonstrate that pharmacological inhibition of the cGAS-STING pathway represents a viable therapeutic approach for mitigating A-T-associated neurodegeneration. Furthermore, it supports the potential utility of STING-targeted therapies in managing other premature aging disorders characterized by self-DNA-driven secretory phenotype activation (Aguado et al., 2021).

### 4.5 Role of STING signaling in Huntington's disease

HD is an autosomal dominant neurodegenerative disorder caused by CAG trinucleotide repeat expansions in the Huntington (HTT) gene. It is characterized by choreiform movements and cognitive decline. Neuropathological studies reveal elevated pro-inflammatory cytokines (IL-6, IL-8, TNF-α) and NF-κB pathway dysregulation in HD patient brains (Träger et al., 2014; Björkqvist et al., 2008). There is evidence that the cGAS-STING-IRF3 signaling pathway is activated in the striatum of HD patients after death. Experimental evidence indicates that melatonin modulates cGAS-STING pathway activation in HD mouse models. Melatonin deficiency disrupts mitochondrial integrity, leading to oxidative stress generation, pathological mtDNA cytoplasmic leakage, and cGAS-STING-dependent IFN production (Jauhari et al., 2020; Miller et al., 2016). The STING protein undergoes p62-mediated ubiquitination in HD, facilitating its sequestration into autophagosomes and subsequent lysosomal degradation (Moretti et al., 2017). Following brief downstream pathway stimulation, cGAS and STING undergo rapid autophagolysosomal degradation (Gui et al., 2019). Inhibition of cGAS in HD striatum cells can reduce autophagy activity and significantly improve cellular inflammatory response (Sharma et al., 2020). Genetic ablation of cGAS attenuates both neuroinflammatory responses and autophagic dysregulation in HD striatal neurons, indicating its pathogenic role in disease progression. These findings position cGAS as a potential therapeutic target for HD intervention. The Toll-interacting protein (TOLLIP) regulates STING homeostasis by stabilizing STING protein under basal conditions. Genetic or functional impairment of TOLLIP substantially decreases STING expression, consequently suppressing DNA-stimulated cGAS-STING pathway activation (Pokatayev et al., 2020). Experimental evidence demonstrates that HTTq74 (PolyQ) protein aggregates are pathologically associated with HD pathogenesis. Cellular overexpression of HTTq74 competitively binds TOLLIP, disrupting the TOLLIP-STING interaction and promoting STING degradation via the lysosomal pathway (Pokatayev et al., 2020). This may effectively inhibit the downstream pathway of STING and reduce the harm caused by HD.

### 4.6 Role of STING signaling in multiple sclerosis

MS represents a progressive neurodegenerative condition affecting the CNS, wherein immune-mediated damage to oligodendrocytes and their myelin membranes results in sustained inflammatory responses and subsequent neuronal fiber deterioration (Hafler, 2004). While the precise etiology of MS remains incompletely understood, acute neuroinflammatory lesions and their subsequent CNS infiltration constitute key drivers of disease progression (Engelhardt and Ransohoff, 2005). Microglial IFN-I gene expression was upregulated in an experimental axon injury model (Mathur et al., 2017; Hosmane et al., 2012). An alternative *in vivo* axonal injury model elicited robust IFN-I responses, wherein impaired IFN-I signaling resulted in exacerbated immune cell infiltration at the lesion site (Khorooshi and Owens, 2010). Immunomodulatory therapies such as IFN and rituximab can prevent or delay the progression of MS (Doshi and Chataway, 2016). Notably, murine studies demonstrate an inverse correlation between ultraviolet radiation exposure and MS incidence. Ultraviolet B irradiation recruits pro-inflammatory monocytes and stimulates IFN-I production through stimulus-responsive mechanisms. Recent investigations reveal that the serine protease inhibitor Bowman-Birk inhibitor ameliorates autoimmune neuroinflammation by promoting STING-dependent IFN-β induction (Casella et al., 2020). A research team reported in Cell a calcium-dependent STING activation mechanism independent of canonical pathways. Their work revealed that during neuroinflammation, IFN-γ induces neuronal STING expression, which remains ER-localized through STIM1 binding. Glutamatergic stimulation triggers STIM1 translocation, enabling STING trafficking to the Golgi apparatus for activation. These findings elucidate how combined IFN-γ and glutamate signaling initiates the neuronal innate immune response in MS and related neurodegenerative disorders (Paul et al., 2021; Jeltema et al., 2023). Collectively, targeting the cGAS-STING axis to stimulate type I interferon production could facilitate novel MS treatment development.

### 4.7 Role of STING signaling in ischemia stroke

IS is characterized by cerebral dysfunction resulting from interrupted or severely diminished blood flow, leading to brain tissue hypoxia. As the predominant stroke subtype, emerging evidence suggests IS may also represent a form of NDs. IS pathogenesis involves multifactorial etiologies and intricate pathophysiological mechanisms. Metabolic dysregulation commonly triggers aberrant cGAS-STING pathway activation across various cell types, with vascular endothelial cells exhibiting particular vulnerability (Huang et al., 2020; Yu et al., 2022). Mitochondrial impairment, particularly when accompanied by aberrant mtDNA leakage, serves as a critical biomarker of metabolic distress. The functional interplay between mitochondrial autophagy and the cGAS-STING axis constitutes a fundamental mechanism underlying neuroinflammatory processes and neurodegenerative pathogenesis (Zhou et al., 2024). The cGAS-STING pathway exacerbates ischemic brain injury. In a murine middle cerebral artery occlusion (MCAO) model, cGAS silencing attenuated microglial M1 polarization, normalized the M1/M2 phenotypic ratio, and mitigated neuroinflammation by modulating microglial activation states (Jiang et al., 2021). Following ischemia-reperfusion injury (IRI), microglial STING pathway activation drives M1 macrophage polarization, fostering a proinflammatory milieu that amplifies cerebral inflammation and worsens IRI outcomes. This study demonstrates that dysregulated iron metabolism critically contributes to IS pathogenesis through oxidative stress mechanisms. During initial ischemia-reperfusion, Nuclear Receptor Coactivator 4 (NCOA4)-dependent ferritinophagy increases free iron levels, triggering oxidative stress that activates both autophagy and apoptosis, ultimately exacerbating neural damage (Li et al., 2021). Systematic analysis of NCOA4-cGAS-STING interactions demonstrates that this signaling cascade worsens ischemic brain injury via NCOA4-driven ferritinophagy (Li et al., 2023). Ferroptosis is executed through mitochondrial voltage-dependent anion channels, with peroxidation of polyunsaturated fatty acids (PUFAs) in cellular membranes constituting a central biochemical feature. Recent experimental evidence indicates that STING signaling modulates PUFA biosynthesis through suppression of Δ6-desaturase activity, the catalytic step mediated by fatty acid desaturase 2 that determines metabolic flux through this pathway (Vila et al., 2022). As previously described, NCOA4 expression is modulated by the upstream cGAS-STING signaling axis, thereby amplifying ferritinophagy-mediated ischemic damage (Li et al., 2023).

### 4.8 Role of STING signaling in traumatic brain injury

TBI results from an initial external force to the brain, followed by a secondary injury. This secondary injury includes molecular, chemical, and inflammatory reactions that cause brain tissue damage and neurological impairments (Galgano et al., 2017). IFNs serve as critical immunomodulators that mediate neuroinflammatory responses following TBI (de Weerd et al., 2007). Attenuation of IFN-I signaling mitigates neuroinflammation and ameliorates neuronal injury in the controlled cortical impact (CCI) murine TBI model (Karve et al., 2016). A research team has pointed out that STING and type I interferon-stimulated genes are upregulated in a biphasic manner after CCI injury. NLRX1 may be an additional regulatory factor that functions upstream to block the activation of STING and thereby regulate the cGAS-STING pathway in the brain (Fritsch et al., 2022). Abdullah and colleagues also demonstrated that STING plays a pivotal role in regulating IFN production and neuroinflammatory cascades following TBI. Genetic ablation of STING (STING^−/−^) conferred neuroprotection in the CCI model, with significantly attenuated cortical damage compared to wild-type controls. Postmortem analyses of TBI patient brains revealed elevated STING transcript levels, substantiating its pathogenic contribution to TBI progression. Emerging evidence proves that the cGAS-STING pathway becomes activated following TBI, with STING-mediated inflammatory responses contributing to both neuroinflammatory processes and the detrimental consequences of impaired autophagy (Abdullah et al., 2018). In another study, GSK2656157 (or PERK knockdown) was used to block PERK phosphorylation, which weakened the entire neuroinflammatory cascade reaction and improved cognitive function after TBI. That is, by inhibiting PERK, the classical pathway mediated by STING and the production of interferons in neurons after TBI were suppressed, thereby reducing brain damage and cell loss (Sen et al., 2020). This suggests that the regulation of the STING pathway is of great help to TBI.

## 5 Targeting STING for the treatment of NDs

### 5.1 The direct inhibitor of STING

**Figure 5:**
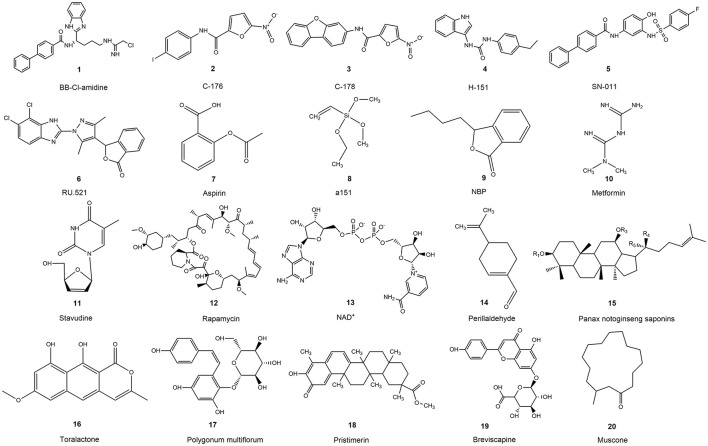
The chemical structural formula of the active ingredients in medicines or Chinese herbal medicines.

Precision modulation of STING signaling may offer superior neuroprotective efficacy in CNS trauma (Li et al., 2013). STING inhibitors can be classified into two principal categories based on their mechanism of action. The first strategy involves targeting the palmitoylated transmembrane domain of STING. The second is to occupy the CDN binding pocket, thereby blocking STING activation. This section outlines these pharmacological approaches and presents representative STING inhibitors. We attach the chemical structure formula of STING small molecule inhibitors and Chinese herbal medicine components in Figure 5 and summarize it in Table 1.

**Table 1:** Drugs for the treatment of NDs through the STING signal pathway.

**Inhibitors**	**Mechanism of action**	**Models**	**Related NDs**	**References**
BB-Cl-amidine	Modifying STING in a PAD-independent manner to impair oligonucleation and downstream pathways	Trex1^D18N/D18N^ mouse model of AGS	AGS	Barasa et al., 2023
Compounds C-176 and C-178	Inhibiting the STING pathway by preventing palmitoylation at Cys91 position	MPTP-induced mouse model of PD, C57BL/6J mice SAH model	PD, TBI, IS	Haag et al., 2018; Wang et al., 2023
H-151	Decreasing TBK1 phosphorylation and inhibit palmitoylation of human STING	cGAS^−/−^, 5 × FAD mice, AppNL-G-F/hTau double-knock-in mice, photothrombotic stroke mouse model	AD, IS	Xie et al., 2023; Chung et al., 2024; Wu et al., 2024
SN-011	Locking STING in an open inactive conformation	C57BL/6J mice Trex1^−/−^ model	AGS	Hong et al., 2021
RU.521	Targeting the active site of cGAS	MPTP-induced C57BL/6J mouse model, BV2 microglial cell	PD, IS, ALS, HI, TBI	Shao et al., 2023; Zhou et al., 2025b
Aspirin	Acetylation of cGAS at three lysine sites inhibits its enzymatic function	Primary human ONS cells, C57BL/6J mice Trex1^−/−^ model	A-T, AGS	Dai et al., 2019
a151	Competing with immune-stimulating DNA	HIE Rice-Vannucci model, Sprague-Dawley (SD) rats tMCAO model	IS	Li et al., 2020
NBP	Suppressing mitochondrial DNA release and attenuating cGAS-STING signaling	SD rats I/R model, C57BL/6 mice PD model, BV2 microglial cells	PD	Liu et al., 2024
Metformin	Reducing mtDNA release	MPTP-induced mouse model of PD	PD	Wang et al., 2024a
Stavudine	Inhibiting LINE-1 reverse transcription	HIE Rice-Vannucci model	HI	Gamdzyk et al., 2020
NAD^+^	Decreasing cGAS-STING activity	APP/PS1 mutant mice	AD	Hou et al., 2021
Rapamycin	Enhances lysosomal activity and mitochondrial function	C57BL/6 mice	PD	Aharon-Peretz et al., 2004; Sidransky et al., 2009; Chia et al., 2021; Wang et al., 2024b
Traditional Chinese medicines				
Perillaldehyde	Inhibiting cGAS activity	Trex1^+/−^ mice	AGS	Chu et al., 2021
Panaxnotoginsengsaponins	Reducing the expressions of cGAS, STING and p-NF-κB/NF-κB	SD rats MCAO model	IS	Wu et al., 2021
Cassiaobtusifolia L	Inhibiting the activation of the mtDNA-mediated STING pathway	SD rats CIRI model	IS	Yun et al., 2021
Polygonummultiflorum	Reducing STING expression	APPswe/PS1dE9 double transgenic AD model mice	AD	Gao et al., 2023
Pristimerin	Suppressing microglial M1 polarization through cGAS-STING pathway inhibition	SD rats MCAO model	IS	Lin et al., 2023
Breviscapine	Inhibiting the cGAS/STING/NLR pathway or cGAS/STING/NLRP3 axis in microglia	SD rats MCAO model, APP/PS1 mouse model	IS, AD	Duan et al., 2023; Zhang et al., 2022b
Muscone	Suppressing the GAS-S'171ng pathway activation	SD rats MCAO model	BI	Ying et al., 2024

#### 5.1.1 STING inhibitors targeting the palmitoylation site

Protein-citrullination is driven by protein-arginine deiminase (PAD) enzymes. Given its potential impact on the subcellular localization of STING, contemporary studies have explored the role of PAD inhibitors in regulating STING-dependent signal transduction (Humphries et al., 2023). BB-Cl-amidine, a prototypical PAD inhibitor, demonstrates potent anti-inflammatory activity across multiple experimental models. The small molecule BB-Cl-amidine demonstrates exceptional target selectivity, effectively inhibiting STING-mediated signal transduction without affecting alternative innate immune mechanisms at concentrations below 1 nM. BB-Cl-amidine covalently modifies C148, C206, C257, and C309 of STING *in vitro*, with C148 being the most critical for oligomerization. *In vivo*, BB-Cl-amidine responds to dsDNA to inhibit type I IFN production, reduces inflammation and pathology in the Trex1D18N/D18N AGS mouse model, and improves survival (Barasa et al., 2023). In contrast, compounds C-176 and C-178—two nitrofuran derivatives obtained through chemical screening inhibit the STING pathway by preventing palmitoylation at Cys91 position (Haag et al., 2018). A team discovered that in the MPTP-induced PD mouse model, STING expression was significantly increased in the SN and striatum compared to controls. Pharmacological inhibition of STING with C-176 reduced inflammation and improved dopaminergic neurodegeneration and neurobehavioral symptoms in the substantia nigrostriatal region (Wang et al., 2023). Another study also confirmed through the investigation of the phenotypic changes of microglia in the subarachnoid hemorrhage (SAH) model that STING can enhance the inflammatory response by promoting the activation and polarization of microglia into the pro-inflammatory M1 phenotype. C-176 treatment can significantly inhibit STING-mediated neuroinflammation after SAH. It was proposed that AMP-activated protein kinase (AMPK) signaling also plays an important role in the neuroprotective effect of C-176, but the exact interaction between STING activation and AMPK inhibition remains to be confirmed (Peng et al., 2020). Through structural optimization of C-176 and C-178, the novel compound H-151 has been developed as a potent and selective covalent STING antagonist with improved pharmacological properties (Haag et al., 2018). This compound suppresses IFN-I synthesis, attenuates TBK1 phosphorylation, and prevents palmitoylation of human STING protein (Liu et al., 2020). Furthermore, administration of H-151, a selective STING pathway inhibitor, successfully suppressed cGAS-STING signaling activation and ameliorated AD-like pathological characteristics in the 5 × FAD mouse model (Xie et al., 2023). At the same time, another research team also demonstrated that STING inhibition by H-151 reduced a wide range of AD pathogenic features in App^NL − G−F^/hTau double-knock-in mice (Chung et al., 2024). They monitored and measured the expression of various AD-related risk factors in dKI microglia, including upregulated aging marker genes (Cdkn1a, Cdkn2a), downstream cGAS-STING pathway genes (Irf7, Stat1, Ifitm3, Cxcl10), and the cGAMP-degrading enzyme Enpp1. Wu et al. (2024) started intraperitoneal injection of H151 for 8 consecutive days 1 h after inducing a photothrombotic stroke model in mice. The results indicated a decrease in the number of SYP or PSD95 phagocytic cells in microglia. Further analysis revealed that after STING inhibition, the mRNA levels of several complement components and phagocytic receptors decreased, and the nuclear translocation of the phosphorylated transcription factor STAT1 was also inhibited in microglia. It is worth noting that Zhou et al. (2025a) designed a series of novel indole derivatives aimed at inhibiting STING, based on the structures of covalent STING inhibitors H151 and C178. Among these new compounds, 4dc exhibited superior potency compared to H151, with IC50 values of 0.14 μM in RAW-LuciaTM ISG cells and 0.39 μM in THP1-Dual™ cells. These results indicate that 4dc demonstrates strong inhibitory activity against STING and holds promise for further investigation.

#### 5.1.2 STING inhibitors targeting CDN binding site

The computational docking analyses have revealed SN-011 as a highly effective STING pathway inhibitor. Mechanistically, SN-011 binds to the CDN-binding pocket with greater affinity than the endogenous cGAS product,2′3′-cGAMP, thereby stabilizing the STING dimer in an open and functionally inactive conformation. SN-011 and H-151 inhibit STING with comparable efficacy in mouse cell lines, exhibiting an IC50 of approximately 100 nM. However, H-151 markedly compromises cell viability and induces cytotoxicity, whereas SN-011 demonstrates no such adverse effects. In Trex1-deficient AGS mouse models, SN-011 administration demonstrated notable therapeutic efficacy in mitigating multi-system damage, particularly neurological impairment (Hong et al., 2021).

### 5.2 The upstream inhibitor of STING

Several structurally similar compounds exert their inhibitory effects on cGAS through competitive binding mechanisms, primarily by occupying the enzyme's active sites that normally interact with ATP, GTP, or the catalytic product cGAMP, or by interfering with the initial activation step of cGAS by interfering with DNA binding to cGAS. Alternative approaches, such as preventing cGAS dimerization, modifying its N-terminal dsDNA binding domain, or indirect modulation via other targets, have been investigated. Key inhibitors are discussed in the next section.

#### 5.2.1 Inhibit the activation of cGAS

The small-molecule compound RU.521 demonstrates potent and selective inhibition of cGAS activity, as evidenced by cellular assays evaluating cGAS-dependent signaling pathways (Vincent et al., 2017). An increase in the expression level of cGAS was observed in the brain tissue of rats after SAH, while the inhibition of cGAS with the specific inhibitor RU.521 led to a decrease in brain water content and the permeability of the blood-brain barrier. Meanwhile, it improved the neurological deficits, cognitive impairment and dendritic spinal density after SAH. This was confirmed to be achieved by regulating microglial polarization and neuroinflammation mediated by the cGAS/STING/NF-κB pathway. RU.521 increased the expression of M2 phenotypic markers Arg-1 and CD206, while reducing the expression of M1 phenotypic markers iNOS and CD16 (Shao et al., 2023). The latest investigations reveal spatial colocalization of IRF7 with microglial populations within the murine PD brain, underscoring critical interactions between IRF7 and the microglial cGAS-STING signaling axis. When BV2 microglial cultures were administered RU.521 at 2 μM concentration for a 12-hour incubation period prior to MPP+ exposure (20 μM), the subsequent disruption of cGAS-STING pathway integrity was accompanied by marked attenuation of IRF7 phosphorylation. This pharmacological intervention implies RU.51's capacity to modulate microglial activation states through IRF7-dependent mechanisms, offering promising therapeutic potential for PD neuropathology (Zhou et al., 2025b). Investigations in both AGS patient-derived cellular models and murine systems demonstrate that aspirin induces post-translational acetylation of cGAS at critical regulatory sites. This covalent modification disrupts cGAS's capacity to initiate downstream innate immune signaling cascades, thereby attenuating autoimmune manifestations. Specifically, aspirin-mediated acetylation targets three distinct lysine residues within the cGAS protein structure, resulting in functional impairment of the enzyme. These molecular alterations correlate with significant anti-inflammatory effects observed in Trex1-deficient rodent models and AGS patient fibroblasts, underscoring the therapeutic potential of pharmacological cGAS acetylation in modulating sterile neuroinflammatory disorders (Dai et al., 2019).

#### 5.2.2 Inhibitors targeting the cGAS-dsDNA interaction

A151 is a synthetic oligodeoxynucleotide designed to antagonize cGAS activity. It exerts neuroprotective effects in IS by competitively binding to cytoplasmic nucleic acid-sensing cGAS and absent in melanoma 2 inflammasome, thereby blocking immunostimulatory DNA recognition. Experimental evidence demonstrates that microglia-specific cGAS deletion markedly attenuates neuroinflammation and mitigates histopathological alterations in rodent models of cerebral ischemia-reperfusion injury. Moreover, pharmacological inhibition of cGAS significantly decreases delayed neuronal apoptosis following IRI (Li et al., 2020). Interestingly, a research team developed a neutrophil-hijacked nanoplatform to reprogram NETosis for ODN delivery and neuroinflammatory regulation. A151 is encapsulated by apoptotic bodies and specifically phagocytosed by microglia. This provides a new drug delivery method, offering a better option for the treatment of inflammatory diseases in the CNS (Yin et al., 2024). Recent research demonstrates that dl-3-n-butylphthalide (NBP) exerts neuroprotective effects by attenuating neuroinflammatory responses and preserving dopaminergic neuron viability. The study on the upregulation of cGAS-STING signaling by rotenone and its induction of inflammatory responses in BV2 cells reveals that NBP not only reduces mitochondrial ROS production and limits cytosolic mtDNA escape but also suppresses pathological microglial overactivation. These findings elucidate a previously unrecognized mechanism underlying NBP's therapeutic effects (Liu et al., 2024). Metformin, a widely prescribed first-line medication for glycemic control (Sanchez-Rangel and Inzucchi, 2017), has recently emerged as a potential therapeutic agent with anti-aging properties and neuroprotective effects in the CNS (Cheng et al., 2022; Kodali et al., 2021; Bharath et al., 2020). Emerging experimental evidence demonstrates that metformin effectively suppresses astrocytic senescence both in cellular models and in murine models of PD. There's evidence that reveals metformin treatment significantly attenuates cellular senescence in astrocytes across both cell culture systems and animal models through regulation of the Mfn2-cGAS axis. Mechanistic studies further elucidate that metformin's therapeutic effects are mediated through Mfn2-dependent mitochondrial homeostasis restoration, resulting in reduced mtDNA leakage into the cytosol. Consequently, this pharmacological intervention effectively inhibits cGAS-STING pathway activation, thereby mitigating astrocyte senescence and potentially slowing PD pathogenesis (Wang et al., 2024a). The STING pathway is activated by cytoplasmic DNA derived from long interspersed Element class 1 (LINE-1). Previous studies have documented increased LINE-1 expression in the striatum and dentate gyrus of adult rats following methamphetamine administration. Enhanced LINE-1 expression was also detected in *in vivo* models of cell death induced by acute oxidative stress and in dopaminergic neurons in the midbrain of adult rats (a type of neuron that degenerated in PD; Moszczynska et al., 2015; Blaudin de Thé et al., 2018). Marcin et al. showed that in a 10-day-old rat model, the cGAS/STING pathway was activated 24–48 h after Hypoxia-Ischemia (HI). STING silencing via siRNA reduced infarct size, cortical neurodegeneration, and improved neurological behavior within 48 h, suggesting that STING contributes to injury progression after HI. Further findings indicate that LINE-1 is a potential upstream activator of this pathway and increases 48 h post-HI. Stavudine, a Nucleoside Reverse Transcriptase Inhibitor (NRTI) that blocks LINE-1, reduced infarct size, cortical neuronal degeneration, and lowered Bax and cleaved caspase 3 expression (Gamdzyk et al., 2020). However, this experiment also has certain limitations because it focuses on the short-term effects of cGAS/STING inhibition in the experimental HI and does not examine the results at a longer time point to confirm the therapeutic effect and evaluate the functional recovery. Nicotinamide adenine dinucleotide (NAD^+^) and its metabolic precursors have been shown to suppress proinflammatory cytokine production and attenuate microglial and astrocytic activation through modulation of the cGAS-STING signaling pathway. Hou and colleagues demonstrated that, administration of nicotinamide riboside, a key NAD^+^ precursor, enhanced mitophagic activity and ameliorated both cognitive performance and synaptic plasticity in transgenic APP/PS1 mice and suppressed cGAS-STING pathway activation. Notably, nicotinamide riboside treatment also facilitated microglial phenotypic switching from the pro-inflammatory M1 state to the neuroprotective M2 state (Hou et al., 2021). Glucocerebrosidase, encoded by the GBA1 gene, is a lysosomal enzyme that breaks down glucosylceramide (GC) into ceramide and glucose (Breiden and Sandhoff, 2019). In individuals with a single GBA mutation, the risk of developing PD and Lewy body dementia increases (Aharon-Peretz et al., 2004; Sidransky et al., 2009; Chia et al., 2021). Glucocerebrosidase deficiency causes pathological GC accumulation in neurons, leading to α-syn aggregation, which impairs Glucocerebrosidase transport from the ER to lysosomes and disrupts its function (Mazzulli et al., 2011). Recently, a research team used primary neurons, microglia, and astrocytes from wild-type C57BL/6 mice cultured *in vitro* to study how GC accumulation affects STING signaling. They found that GC accumulation in microglia causes mtDNA to leak into the cytosol, triggering STING-dependent inflammation. Additionally, GC-induced lysosomal damage impairs the degradation of activated STING. Rapamycin, which enhances lysosomal activity and mitochondrial function, can reduce STING signaling (Wang et al., 2024b). In addition, there are the latest drugs recently. For example, Yan's team discovered that Berbamine targets STING, resulting in the down-regulation of p-STING (Ser366) and CCL2 (Yan et al., 2025). Zhao et al. (2025) developed a small-molecule STING inhibitor and a STING mutant-specific degrader by targeting the two coupled pockets of the STING dimer. Compounds SI-24, SI-42, and SI-43 effectively inhibited STING activation and the subsequent release of IFN-β and CXCL-10 induced by2′3′-cGAMP. However, due to space limitations and whether cytological and zoological experiments related to neuroinflammation were conducted, the review did not elaborate specifically. Overall, drugs and methods that can inhibit the function of STING itself, the activation of cGAS, or the activation of other non-classical STING pathways may all inhibit the activation of downstream signaling pathways of STING. However, as STING plays a significant role in immune regulation, how to achieve precise targeting of this pathway in NDs to exert therapeutic effects and minimize the impact on normal immune function remains an important direction in new drug development. Current drug development is mainly based on known targets and enhances performance by optimizing the structure of existing active derivatives. Future studies can explore more potential regulatory pathways and intervene in other key nodes in the STING signaling pathway, with the aim of improving therapeutic effects and reducing toxic side effects.

### 5.3 Compounds from traditional Chinese medicine acting on STING

The traditional Chinese herbal medicines have not explained much about the cGAS-STING pathway in NDs, but the relevant pharmacological mechanisms and pathways are abundant. Next, we will discuss some TCMs covering the cGAS-STING pathway. Chu et al. (2021) reported that perillaldehyde inhibits cytoplasmic DNA induced innate immunity by inhibiting cGAS activity. Volatile monoterpenoid perilla aldehyde (PAH) is the main and most effective component of perilla leaves. Studies have demonstrated that PAH specifically impairs innate signal transduction and inflammatory responses induced by cytoplasmic DNA by inhibiting the activity of cGAS. The AGS disease model treated with perillaldehyde—a Trex1 gene knockout mouse showed reduced expression levels of multiple interferon ISG such as Cxcl10, Isg15, Isg56, and Jfit3, and markedly attenuated autoimmune inflammatory responses. Since PAH shows inhibitory effects on cGAS in both mice and humans, the specific mechanism by which PAH structurally disrupts the binding of cGAS to dsDNA or acts on the conservative active site of cGAS still needs to be determined. Panaxnotoginsengsaponins (PNS) injection has the effect of promoting blood circulation and removing blood stasis, adjusting unclogged arteries (Bumblauskiene et al., 2009). Current clinical practice has established the safety and efficacy of acute cerebral infarction management. The study detected the levels of TNF-a and IL-6 in the cortex and the iNOS gene table. It was found that the protein expressions of CGAS and STING in the cortical tissues of MCAO rats significantly increased, and the protein expressions of p-NF-κB/NF-κB also increased. PNS effectively inhibited the cGAS/STING pathway. That is, PNS may inhibit the inflammatory response induced by NF-κB by regulating the cGAS/STING pathway (Wu et al., 2021). Similarly, Cassiaobtusifolia L is a traditional Chinese medicine that is traditionally used for its hepatoprotective properties, to improve vision, promote diuresis, and relieve constipation. The toralactone is a possible effective medicinal ingredient. Studies have found that Cassiaobtusifolial L can reduce the degree of edema in rats with TBI, promote the recovery of nerve function, and have brain protection (Qiao et al., 2020). In the study, after treatment with an aqueous extract of Semen Cassiate, the mtDNA level and the protein levels of p-STING/STING and cGAS in the cytoplasm of the cortical area were significantly decreased, indicating that Semen Cassiate could inhibit the activation of the mtDNA-mediated STING pathway, thereby alleviating brain damage in cerebral ischemia/reperfusion injury (CIRI) rats (Yun et al., 2021). Tetrahydroxy stilbene glucoside (TSG), the primary bioactive constituent derived from Polygonum multiflorum, has been the focus of recent investigations into cognitive function and neuroinflammatory modulation. Experimental studies utilizing the APPswe/PS1dE9 transgenic AD mouse model demonstrated that TSG administration significantly downregulated hippocampal NLRP3 inflammasome expression. At the same time, Cell culture experiments showed that TSG reversed LPS- and IFN-γ-induced M1 microglial activation toward a quiescent state. TSG also normalized the increased cGAS-STING levels observed in activated microglia (Gao et al., 2023). Pristimerin is a quinone formamide triterpene compound extracted from Celastraceae and Portulacaceae. Monitoring of the expression of corresponding substances in the MCAO rat model suggests that it can inhibit the expression of cGAS and STING proteins, down-regulate the levels of IL-6 and IL-1β, and thereby alleviate the pathological development of brain tissue in rats. That is to say, after PT intervention, the expression of iNOS in the brain tissue of rats with ischemic stroke was down-regulated, the expression of Arg-1 was up-regulated, and the levels of pro-inflammatory factors IL-6 and IL-1β were decreased. The findings imply that the therapeutic mechanism may involve suppression of microglial polarization toward the proinflammatory M1 phenotype via modulation of the cGAS/STING signaling cascade, ultimately ameliorating stroke-induced cognitive deficits and neuroinflammation (Lin et al., 2023). Of course, its specific mechanism still awaits further research. Breviscapine, a bioactive flavonoid derived from Erigeron breviscapinus, exhibits multi-target pharmacological properties including antioxidative, anti-inflammatory, and vascular endothelial modulatory effects. Experimental investigations by Duan et al. employing MCAO rodent models demonstrated that scutellarin (the primary active constituent of breviscapine) confers neuroprotection against cerebral ischemia through suppression of microglial cGAS-STING-NLRP3 inflammasome signaling. Furthermore, their research revealed this compound's potential to ameliorate Alzheimer's-related cognitive deficits via modulation of the PKA/CREB/HDA3 transcriptional regulation pathway (Duan et al., 2023; Zhang et al., 2022b). Muscone is the main component of musk in TCMs, which has anti-inflammatory, anti-oxidation, anti-apoptosis, and neuroprotective effects. In the Oxygen-Glucose Deprivation/Reperfusion induced PC12 cell model, musk ketone can enhance cell viability and inhibit apoptosis through the Bax/Bcl-2/Caspase-3 pathway. Notably, muscone administration was shown to significantly downregulate the expression of these pathway components. The observed neuroprotective effects appear to be mediated through suppression of cGAS-STING pathway activation, thereby attenuating cerebral ischemic damage and associated neuroinflammatory responses. These findings were further substantiated through systematic validation of muscone's modulatory effects on this signaling cascade. But the specific mechanism remains to be explored (Ying et al., 2024).

## 6 Conclusion

In NDs, STING is activated by multiple upstream pathways and regulates inflammation, autophagy, and other related pathophysiological reactions in the nervous system through downstream pathway regulation. The involvement of STING-related pathways in various NDs and acute neurological disorders has been established. Modulating STING can effectively suppress inflammatory responses and mitigate brain tissue damage in these conditions. The activation of STING and metabolic pathways such as ferroptosis, as well as its relationship with microglia and astrocytes, have gradually emerged in the new research progress Currently, there is ongoing development of drugs targeting the STING pathway; however, at present, the development of new drugs is mainly based on the discovered action sites and extensive dsDNA leakage. The point that targeted therapy for neurodegeneration can avoid damage to normal immune function still needs to be further explored Chinese herbs possess extensive medicinal and commercial value within China's healthcare system. The development of new drugs that are affordable and have few side effects and target and regulate the STING pathway will undoubtedly have a positive impact on human health.
